# Ambient Electromagnetic Wave Energy Harvesting Using Human Body Antenna for Wearable Sensors

**DOI:** 10.3390/s25154689

**Published:** 2025-07-29

**Authors:** Dairoku Muramatsu, Kazuki Amano

**Affiliations:** Department of Mechanical and Intelligent Systems Engineering, The University of Electro-Communications, 1-5-1 Chofugaoka, Chofu 182-8585, Japan

**Keywords:** wearable sensor, energy harvesting, ambient electromagnetic wave, human body antenna

## Abstract

Wearable sensors are central to health-monitoring systems, but the limited capacity of compact batteries poses a challenge for long-term and maintenance-free operation. In this study, we investigated ambient electromagnetic wave (AEMW) energy harvesting using a human body antenna (HBA) as a means to supply power to wearable sensors. The power density and frequency distribution of AEMWs were measured in diverse indoor, outdoor, and basement environments. We designed and fabricated a flexible HBA–circuit interface electrode, optimized for broadband impedance matching when worn on the body. Experimental comparisons using a simulated AEMW source demonstrated that the HBA outperformed a conventional small whip antenna, particularly at frequencies below 300 MHz. Furthermore, the outdoor measurements indicated that the power harvested by the HBA was estimated to be −31.9 dBm (0.64 μW), which is sufficient for the intermittent operation of low-power wearable sensors and Bluetooth Low Energy modules. The electromagnetic safety was also evaluated through numerical analysis, and the specific absorption rate was confirmed to be well below the international safety limits. These findings indicate that HBA-based AEMW energy harvesting provides a practical and promising approach to achieving battery-maintenance-free wearable devices.

## 1. Introduction

**Figure 1:**
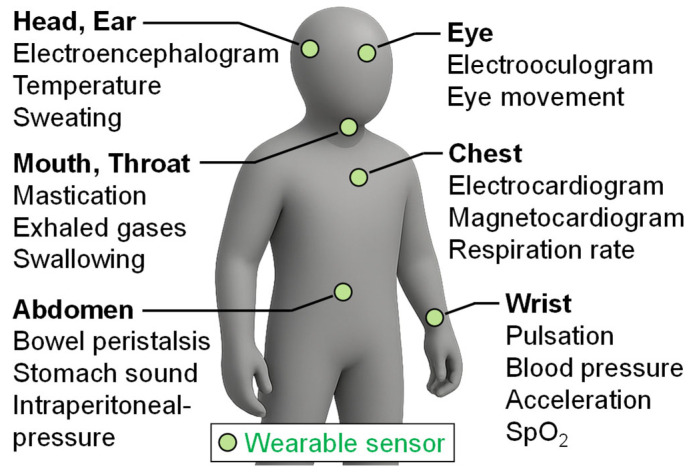
Integrated daily healthcare system supported by multiple wearable sensors.

With the innovation of semiconductor process technology, high-density integration, and advanced materials, information and communication devices as well as sensors have become remarkably smaller, lighter, and more power-efficient. As a result, it has become commonplace for users to live with wearable sensors that can measure various biological signals or wearable gadgets equipped with such sensors. The widespread adoption of wearable sensors has had a significant impact on the medical and healthcare fields. For example, in an integrated daily healthcare system as shown in Figure 1, it is envisioned that wearable sensors would continuously monitor the pulse, body temperature, acceleration, blood oxygen saturation, and other parameters 24 h a day, 365 days a year [1]. The biological signals measured by each sensor are shared over a wireless communication network among the sensors and are ultimately aggregated by a hub device such as a smartphone or smartwatch, enabling the comprehensive monitoring of the user’s health status [2,3,4].

In such a healthcare system, not only is it essential for the sensors to operate continuously, but also for the collected data and control information to be exchanged as needed between sensors and hub devices [5,6,7]. Therefore, extending the operating time of devices and reducing the frequency of recharging are important challenges [8,9,10]. On the other hand, it is desirable for wearable sensors and devices to be small and lightweight, which creates the dilemma of a limited battery capacity. To resolve this dilemma, energy harvesting—which draws electrical power from various environmental sources such as light, heat, and vibration and uses it to drive low-power sensors—has attracted considerable attention [11,12,13].

Environmental power generation by sunlight can generate relatively large amounts of power, depending on the amount of sunlight available [14,15]. For wearable sensor applications, an ultra-flexible energy-harvesting and storage system integrating high-performance organic solar cells and zinc-ion batteries has been reported, achieving a power conversion efficiency of over 16% and a generation output of 10.2 mW/cm^2^ [16]. In addition, for thermal energy harvesting, a wearable thermoelectric generator module using Mg-based thermoelectric materials has been proposed, with a reported output of 18.4 μW/cm^2^ [17]. It has also been demonstrated that, in electromagnetic induction-type power generation, where a small coil inside a wearable device is moved relatively by the user’s motion, generation of up to the mW level is possible [18].

Meanwhile, in a healthcare system that continuously monitors the user’s health status over long periods, it is important not only to have powerful, instantaneous power generation but also to ensure a stable supply of generated power that does not depend on the environment in which the sensors or user is located. Therefore, environmental sources such as sunlight, heat, and vibration, whose output greatly depends on the ambient temperature, the sunlight level, the body location where the sensor is worn, or the user’s activity, present challenges as power sources for wearable sensors [19,20,21].

Of particular interest to us is energy harvesting that uses an antenna to collect and rectify various ambient electromagnetic waves (AEMWs) present in the environment as a power source. In our living environment, there are many systems that utilize electromagnetic waves, including radio and TV broadcasting, Wi-Fi and Bluetooth short-range communications, mobile wireless communications such as smartphones, amateur radio, various public wireless systems, and meteorological or air traffic control radars. There have been attempts to use the AEMW emitted by such systems in our living space as a power source to drive various sensors. Previous studies have included estimations of power recovery near mobile base stations and in indoor environments [22,23], prototype environmental power-generation circuits using television broadcast waves [24], the design of highly efficient rectennas by inkjet printing [25], and the development of a 2.4 GHz rectenna that operates stably even at low input power levels around −30 dBm [26]. However, a major and common issue among previous studies is that antennas of a size suitable for integration into compact wearable devices can harvest only extremely small amounts of power from AEMWs, and only in limited frequency bands.

In this study, we regarded the human body itself, which possesses the properties of both a conductor and a dielectric, as a large antenna element for receiving electromagnetic waves—namely, a human body antenna (HBA)—and investigated a method for dramatically increasing the amount of power harvested from AEMWs to supply wearable sensors. In Section 2, we measure the power density of AEMWs in daily living spaces and determine their frequency distribution and guidelines for energy harvesting. In Section 3, we examine antenna structures suitable for energy harvesting based on the obtained power density distribution of AEMWs and describe the principles of the proposed HBA approach. Section 4 compares and evaluates the signal strengths received by the HBA and a small antenna using a simulated AEMW source and then experimentally estimates the amount of power that an HBA can harvest in an actual environment. Section 5 presents the conclusions. Throughout, we clarify the feasibility of energy harvesting from AEMWs using an HBA.

In addition to the above, the unique contributions of this study are as follows. First, we systematically measured the AEMW power density over a wide range of real-world environments, including indoor, outdoor, and basement settings, which has not been comprehensively addressed in previous studies. Second, we designed and experimentally optimized a flexible wearable interface electrode specifically for HBA applications, focusing on wideband impedance matching. Third, we quantitatively evaluated the harvested power from actual AEMW sources and discussed the practical potential for battery-free wearable sensor systems. These points clarify the innovation and practical significance of our work compared to earlier research.

## 2. Power Density Distribution of AEMWs

### 2.1. Measurement Setup for AEMWs

**Figure 2:**
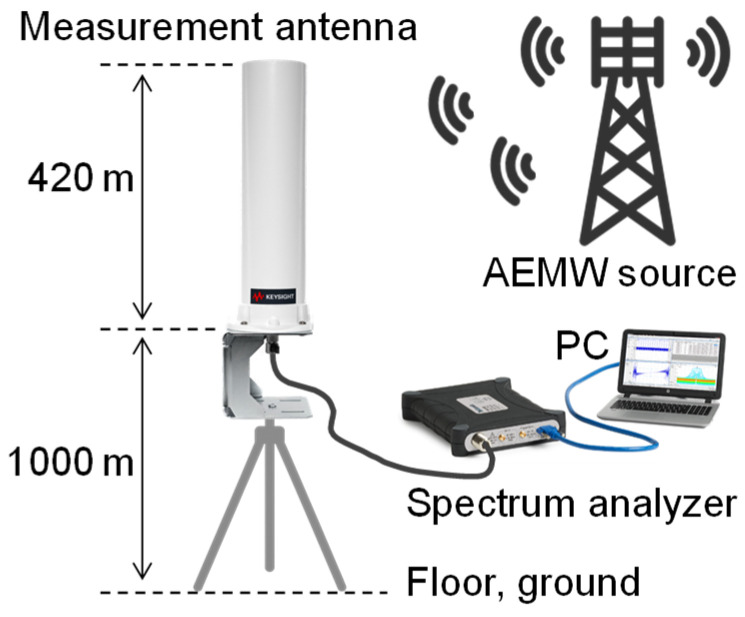
Measurement setup for AEMWs using a standard antenna and spectrum analyzer.

To estimate the amount of power that can be harvested from AEMWs, we first clarified the frequency distribution and environmental dependence of the power density of the electromagnetic waves present in our living space. The power density of AEMWs was measured using a broadband omnidirectional standard antenna (Keysight N6850A, Santa Rosa, CA, USA) and a handheld spectrum analyzer (Tektronix RSA306B, Beaverton, OR, USA). The measurement setup is shown in Figure 2. The standard antenna was 420 mm in total length, covered a frequency range of 20 MHz to 6 GHz, and had an omnidirectional radiation pattern in the horizontal plane. To ensure consistency with the subsequent experiments using the HBA described in the following sections, the height from the floor or ground to the feed point of the standard antenna was set to 1000 mm, corresponding to the wrist position of a standing adult male. In order to accurately capture even the minute power density of AEMWs, the resolution bandwidth of the spectrum analyzer was set to 100 Hz to sufficiently lower the noise floor, and the video bandwidth was set to 30 Hz to average out noise fluctuations. To broadly observe AEMWs in our living environment, the measurement frequency range was set to 30 MHz to 3 GHz [27]. Measurements were performed over five minutes to obtain a sufficiently time-averaged spectrum of AEMWs, and the average value over the entire period was adopted as the result.

The parameter that can be directly measured by the spectrum analyzer is the received power at the standard antenna, so it is necessary to convert the antenna’s received power to the power density at the reception point. The power density *S* at the reception point can be expressed as the ratio of the received antenna power *P*_r_ to the effective aperture *A*_e_, as follows:

(1) $$S=\frac{P_{r}}{A_{e}}$$

The effective aperture *A*_e_ is given by the following formula using the gain *G*_r_ of the receiving antenna and the wavelength *λ* of the electromagnetic wave:

(2) $$A_{e}=\frac{G_{r}\lambda^{2}}{4\pi }$$

Accordingly, the power density *S* can be calculated as follows:

(3) $$S=\frac{4\pi P_{r}}{G_{r}\lambda^{2}}$$

**Figure 3:**
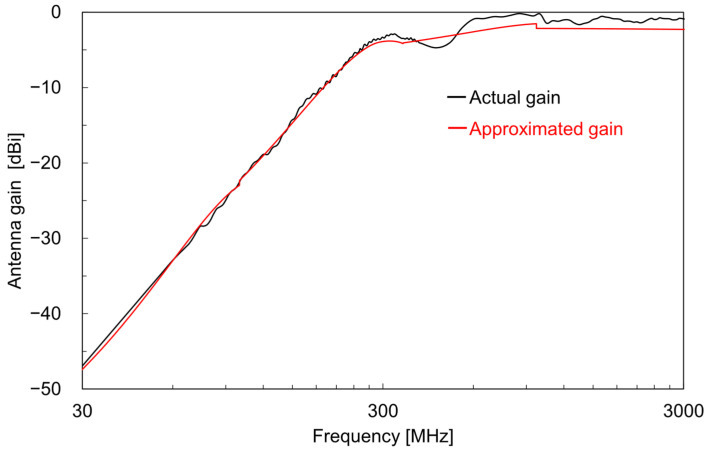
Actual and approximated gain characteristics of the standard antenna.

In this study, the power density *S* at the reception point corresponds to the power density of the AEMW, *P*_r_ corresponds to the power measured by the spectrum analyzer, *G*_r_ is the gain of the standard antenna used for AEMW reception, and *λ* is the wavelength of the AEMW. The frequency characteristics of the gain of the standard antenna used in this study are known at discrete frequencies [28]. Therefore, the antenna gain was approximated by a polynomial so that the power density at any frequency could be calculated. Figure 3 shows the actual and approximated gain characteristics of the antenna. The maximum error between the actual and approximated gains over the entire frequency band was 1.6 dB, confirming that the approximation was sufficiently accurate.

### 2.2. Power Density Spectrum of AEMWs

**Figure 4:**
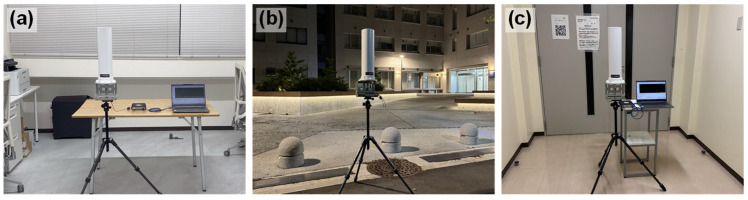
Measurement environments for AEMWs. (**a**) Indoors, (**b**) outdoors, and (**c**) in the basement.

The power density and frequency distribution of AEMWs are expected to differ depending on factors such as the distance from the transmitting antenna and the presence or absence of obstacles in the environment. In other words, the amount of harvested energy may change depending on the environment where the user with the wearable sensor is located. In this study, to confirm the location dependence of AEMWs, measurements of the power density of AEMWs were conducted in three locations on a university campus using the measurement setup described in Section 2.1, as shown in Figure 4: (a) a laboratory on the fifth floor (indoor), (b) a plaza (outdoor), and (c) a corridor on the first basement floor (basement).

**Figure 5:**
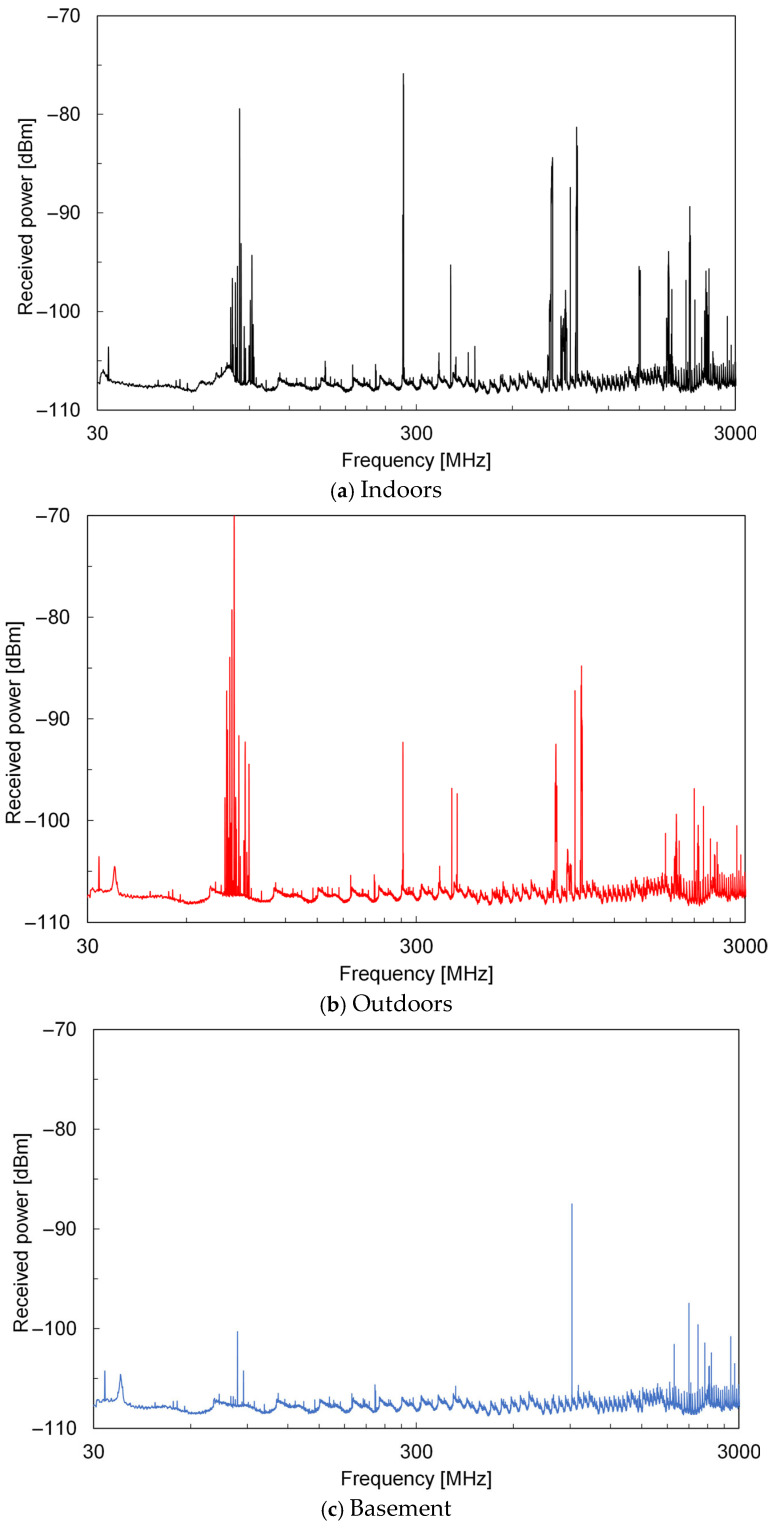
Power spectrum of AEMWs in each measurement environment.

Figure 5 shows the frequency spectrum of the received power of the AEMWs measured at each location. The vertical axis corresponds to the received power *P*_r_, which was used in the calculation of the power density, as shown in Equation (3). In both indoor and outdoor environments, AEMWs were received over a wide frequency range of 30 MHz to 3 GHz. In contrast, in the basement, significant received power was observed only at very limited frequencies compared to the other two locations. This was attributed to strong shielding effects from concrete structures, rebar, and embedded piping in the basement, which make it difficult for electromagnetic waves from above ground to propagate across a wide frequency range.

**Figure 6:**
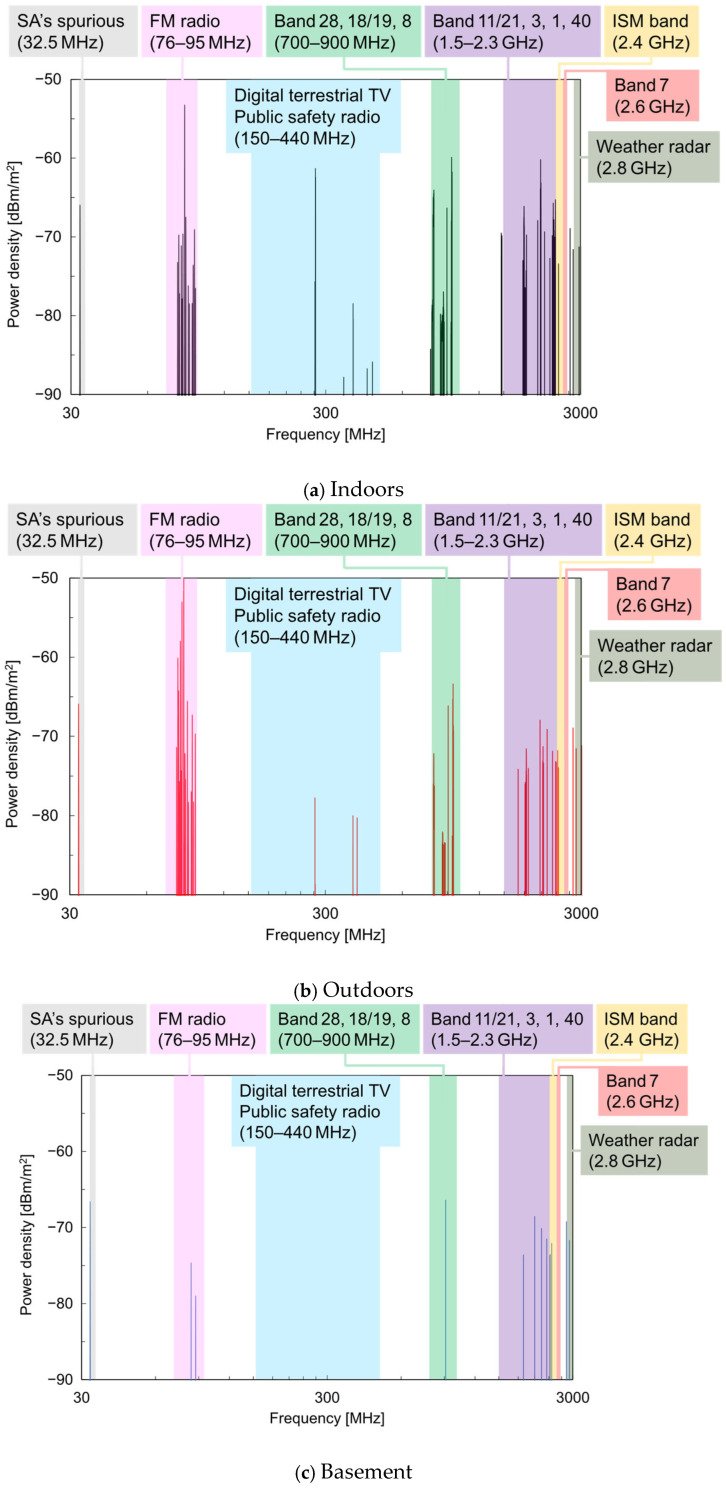
Power density spectrum of AEMWs in each measurement environment.

Next, using Equation (3) and the gain characteristics of the standard antenna shown in Figure 3, the received power *P*_r_ was converted to the power density *S*, and its frequency spectrum is shown in Figure 6. The main wireless systems used in each frequency band are also indicated in the figure. To enhance the visibility of the major power density spectra, if the received power *P*_r_ was below the threshold value *P*th, the power density was set to zero according to the following rule.

(4) $$S=\left\{\begin{array}{cc} \frac{4\pi P_{r}}{G_{r}\lambda^{2}}, & P_{r}>P_{th}\\ 0, & P_{r}\leq P_{th} \end{array}\right.$$

In this study, *P*th was set to −104 dBm based on the power values near the noise floor of the measurement. Compared with the power spectrum in Figure 5, the power density spectrum in Figure 6 shows that, at each location, the relative intensity of the spectrum increased at both low and high frequencies. This is because, at low frequencies, the gain *G*_r_ of the standard antenna was small, and at high frequencies, the wavelength *λ* of the electromagnetic wave was short, resulting in an increased power density *S* according to Equation (3).

Focusing on the differences in the power density spectrum depending on the measurement location, as with the power spectrum, a broad distribution was observed over the measurement range in both the indoor and outdoor locations. In contrast, in the basement, significant power density spectra were observed only near 32 MHz, 1890 MHz, and 2426 MHz. The 1890 MHz peak was believed to be the signal between the base and handset of a digital cordless phone installed in the basement, and the 2426 MHz peak was considered to be the signal from a campus wireless LAN router. The peak observed near 32 MHz was attributed to spurious signals originating from the internal conversion system of the spectrum analyzer, rather than from the actual wireless systems, as its intensity did not change between the indoor, outdoor, and basement environments.

From these results, it was confirmed that, except for special propagation environments such as the basement, AEMWs exist over a wide frequency range both indoors and outdoors. To increase the amount of power generated from AEMW harvesting, it is important to use an antenna capable of efficiently receiving AEMWs with a high power density, particularly in frequency bands below 1 GHz.

## 3. Principle of HBA and Energy-Harvesting Interface

### 3.1. Antenna Structures Suitable for Energy Harvesting

As clarified in Section 2, AEMWs are widely distributed from several tens of MHz to several GHz, with a particularly large power density below 1 GHz. Therefore, to increase the amount of power generated from AEMWs, it is essential to have an antenna that can efficiently receive AEMWs in frequency bands below 1 GHz. The most basic antennas, such as dipole and monopole antennas, are very easy to design to meet the required specifications, such as the resonance frequency, and generally have a relatively high antenna gain. On the other hand, their large and three-dimensional structures make them difficult to implement as compact wearable devices. For example, if a wearable device has an available installation space of 30 mm, the maximum element length (*λ*/2) of an implementable dipole antenna is also 30 mm. The resonance frequency of such a dipole antenna, assuming free space, would be as high as 5 GHz. Dipole antennas can be miniaturized to some extent by loading inductance in the element or folding the structure [29], but increasing the mounting area is unavoidable, making them impractical. In addition, even with inventive mounting methods, it is extremely difficult by resonance principles to shift the resonance frequency down to the MHz band.

Planar antennas are the primary candidates for use in environments with limited installation space [30]. Patch antennas, which are representative of planar antennas, are easy to feed with microstrip lines, allow the integration of the radiation and feeding systems for miniaturization, and are also suitable for low-cost mass production using printed circuit board technology. However, when the antenna becomes electrically smaller than its resonant size, there is a significant drawback in that the radiation resistance drastically decreases. Therefore, if a patch antenna of a size that can be installed in a wearable device is operated in the MHz band, both the antenna gain and the radiation efficiency inevitably deteriorate significantly.

The received power *P*_r_ of AEMWs is given by the Friis transmission equation as follows:

(5) $$P_{r}=P_{t}G_{t}G_{r}{\left(\frac{\lambda }{4\pi r}\right)}^{2}$$

Here, *P*_t_ is the transmitted power of the AEMW source, *G*_t_ is the gain of the transmitting antenna, *G*_r_ is the gain of the receiving antenna for energy harvesting, *λ* is the wavelength of the AEMW, and *r* is the distance between the transmitting and receiving antennas. To increase *P*_r_ and thus increase the generated power, it is desirable to increase *P*_t_, *G*_t_, *G*_r_, and *λ* or to decrease *r*. However, of these parameters, only *G*_r_ can be controlled by the user, so any reduction in the receiving antenna gain directly leads to a decrease in the generated power. From the above, it is difficult to efficiently receive AEMWs in frequency bands below 1 GHz with a small antenna [29], and to increase the amount of power generated from AEMWs, a large antenna of several tens of centimeters to several meters is necessary.

### 3.2. Principle of the HBA

In contrast to small antennas of several tens of millimeters that can be implemented in wearable devices, the human body has an extremely large physical and electrical size. For example, considering a 170 cm tall human body standing on the ground as a quarter-wavelength monopole antenna, its resonance frequency is about 44 MHz. In practice, the human body’s uniquely high dielectric constant causes the electromagnetic wavelength to be shortened. Therefore, electrically, the human body can be considered a larger antenna than its physical size would suggest, and the effective resonance frequency may shift as low as several MHz [31,32]. The possibility that the human body can operate as an antenna—that is, as a human body antenna (HBA)—has been confirmed through numerous analyses and experiments [33,34]. In reality, it is not only the tallest part of the body (height) that functions as an antenna element, but also the arms, legs, and other parts, which can act as independent antenna elements with varying gain. In other words, the human body can partially resonate at various frequency bands, functioning as a multi-band antenna with sufficient potential to broadly receive AEMWs with a high power density in the MHz range. In this study, we focused on such characteristics of an HBA and investigated whether it is possible to receive AEMWs at various frequencies and increase the amount of harvested energy.

Several basic studies have already been conducted on using the human body as an antenna element. For example, it has been reported that, by exciting a communication signal via electrodes attached to the arm, the human body can function as an antenna with an omnidirectional radiation pattern, and this is expected to form a new communication method for body sensor networks [35]. For energy-harvesting applications, it has been reported that a human standing on an aluminum sheet (pseudo-ground) can function as a monopole antenna element [36,37]. There are also studies that have attempted energy harvesting via the human body by utilizing this phenomenon [38]. However, in these studies, an insulator was placed between the pseudo-ground and the feet, so the measurements and energy harvesting were inevitably limited to the feet, making it unsuitable for powering wearable sensors expected to be worn at various locations.

Although only a few studies have assumed energy harvesting using the human body as an antenna from locations other than the feet, some have considered AEMW sources such as fluorescent lamps or LED lighting fixtures and evaluated energy harvesting via wristband-type devices attached to the wrists of subjects standing in close proximity to such fixtures [39,40]. Another study estimated the amount of power harvested in a real environment via an oscilloscope measurement for extending the smartphone operating time [41]. While these studies have demonstrated the possibility of utilizing the human body as an antenna, they were limited to special environments such as near lighting fixtures or outdoors, and they considered only frequency bands below 1 MHz. In contrast, our study provides a comprehensive analysis over a broad frequency range and diverse environments using a wearable-oriented HBA design, as well as a quantitative comparison with a conventional small antenna in realistic settings.

In particular, to properly harvest and rectify the power received by an HBA and use it to supply wearable sensors, it is essential to have an interface that appropriately collects the current induced on the body surface by the received electromagnetic waves [42]. Such an interface must not only be biocompatible and safe but also control the input impedance to collect electrical signals in the desired frequency band without reflection when attached to the human body [43].

### 3.3. Prototype and Evaluation of Energy-Harvesting Interface

**Figure 7:**
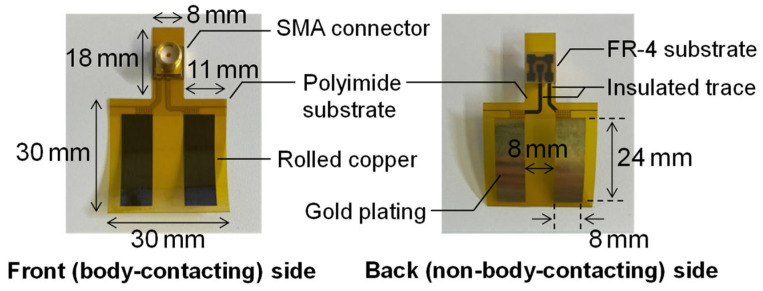
Prototype interface electrode for energy harvesting.

**Figure 8:**
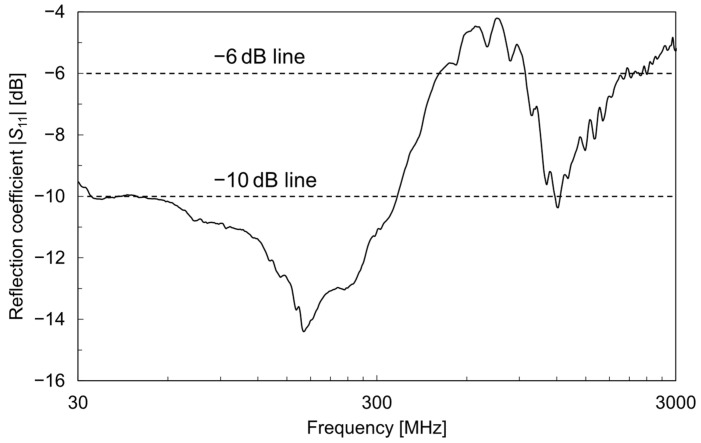
Reflection coefficient of the interface electrode worn on the subject’s wrist.

The prototype interface electrode for energy harvesting between an HBA and the circuit developed by the authors is shown in Figure 7. This interface electrode consists of a flexible polyimide substrate and a rolled copper pattern with biocompatible gold plating and can be closely attached to various parts of the body and reused repeatedly. The dimensions of the polyimide substrate are 30 mm × 30 mm, with an 8 mm × 18 mm extension for mounting the SMA connector. Each electrode pattern measures 8 mm × 24 mm and is arranged at an 8 mm interval. The electrodes and the SMA connector are connected by copper traces with insulated surfaces, and the rear side of the connector is reinforced with an FR-4 dielectric substrate. The polyimide substrate is 25 μm thick, and the copper pattern with plating is 20 μm thick. Assuming implementation in a wearable device such as a smartwatch, the reflection coefficient when the interface electrode is attached to the wrist was measured using a vector network analyzer (E5080A, Keysight, Santa Rosa, CA, USA). Figure 8 shows the frequency characteristics of the measured reflection coefficient. Among the AEMW frequency range of 30 MHz–3 GHz clarified in Section 2, the reflection coefficient remained below −10 dB up to about 350 MHz, indicating that the prototype interface electrode has excellent low-reflection characteristics. Even above 350 MHz, the reflection coefficient was below −6 dB for most of the range, realizing practically sufficient matching. In the 920 MHz–2 GHz range, the reflection coefficient degraded to about −4.2 dB, but the impact of this mismatch was equivalent to about 2 dB in transmission characteristics, which is sufficiently acceptable for practical use. In the following investigations, measurements were conducted with the interface electrode attached to the wrist, assuming implementation in a wearable device such as a smartwatch.

## 4. Evaluation of Reception Characteristics and Harvested Power of HBA

### 4.1. Evaluation of HBA Reception Characteristics Using Simulated AEMW Source

**Figure 9:**
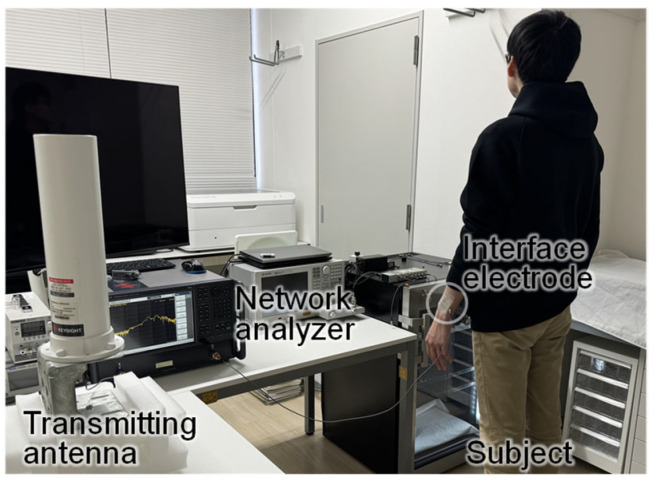
Measurement setup for the reception characteristics of an HBA using a simulated AEMW source.

The interface electrode described in Section 3.3 was attached to the left wrist of a Japanese adult male subject (height: 178 cm), and a simulated AEMW source was placed in proximity to the subject to evaluate the reception characteristics of the HBA. The measurement setup is shown in Figure 9. A broadband omnidirectional standard antenna (Keysight N6850A, Santa Rosa, CA, USA) was used as the transmitting antenna for the simulated AEMWs. The distance between the subject and the standard antenna was 1 m. Port 1 of the vector network analyzer (E5080A, Keysight, Santa Rosa, CA, USA) was connected to the standard antenna, and Port 2 was connected to the interface electrode worn by the subject. The transmission characteristics between the ports were measured as the reception characteristics of the HBA. For comparison, the reception characteristics of a small 2.4 GHz whip antenna (W1095, Pulse Electronics, Inc., San Diego, CA, USA) were also measured using the same setup. The small antenna (excluding the connector part) was 41.5 mm in length and 8 mm in diameter, with a gain of 1 dBi.

**Figure 10:**
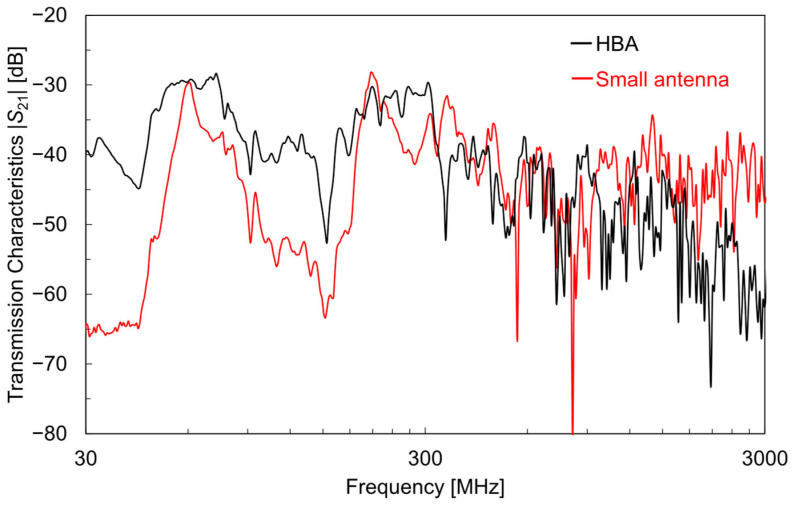
Reception characteristics of an HBA and a small antenna using a simulated AEMW source.

Figure 10 shows the measurement results of the antenna reception characteristics using the simulated AEMW source. The black line indicates the HBA, and the red line indicates the small 2.4 GHz antenna. The HBA showed much better reception characteristics than the small antenna in frequency bands below 300 MHz. This is considered to be because an HBA functions as an electrically large antenna element and efficiently receives simulated AEMWs in the MHz band. However, near 64 MHz and 210 MHz, the reception characteristics of the small antenna were approximately −30 dB, similar to the HBA. These reception characteristic peaks were not due to the small antenna element itself but were believed to be caused by the coaxial cable acting as an antenna and radiation due to common-mode current. It was confirmed that these peak frequencies shift when the cable length is changed, so such reception peaks are not expected to occur when the small antenna is actually implemented in a wearable device.

On the other hand, in frequency bands above 300 MHz, the superiority of the reception characteristics between the HBA and the small antenna varied depending on the frequency. Notably, near 2.4 GHz, the small antenna, which is specifically designed for that frequency band, showed superior reception characteristics. In contrast, the HBA exhibited overall deteriorating reception characteristics at higher frequencies, although there were some small fluctuations. This was mainly because the received electromagnetic waves were increasingly absorbed and lost in the body’s water content at higher frequencies [44]. Therefore, for energy harvesting in specific GHz frequency bands, small antennas are suitable, whereas for broadband energy harvesting, including MHz bands, an HBA is advantageous.

### 4.2. Estimation of Harvested Power of HBA in Actual Environment

In the previous section, the reception characteristics of an HBA were evaluated using a simulated AEMW source. In this section, we estimate the amount of power harvested by an HBA in an actual environment over the frequency range of 30 MHz–3 GHz. The estimation was performed outdoors on a university campus plaza, where Section 2.2 showed that the power density spectrum of the AEMWs was particularly strong near 80 MHz. The interface electrode attached to the subject’s left wrist was connected to a handheld spectrum analyzer (Tektronix RSA306B, Beaverton, OR, USA), and the frequency spectrum of the power received by the HBA was measured from 30 MHz to 3 GHz. The total power *P*_sum_ that can be harvested by the HBA was calculated using the following formula:

(6) $$P_{sum}=\frac{\Delta f}{B}\sum_{k=1}^{n}P_{k}$$

Here, Δ*f* is the measurement frequency interval of the spectrum analyzer, *B* is the resolution bandwidth, *P*_k_ is the received power at the *k*-th measurement frequency point, and *n* is the total number of measurement frequency points. In this study, Δ*f* = 93.4 kHz, *B* = 100 Hz, and *n* = 31,785.

**Figure 11:**
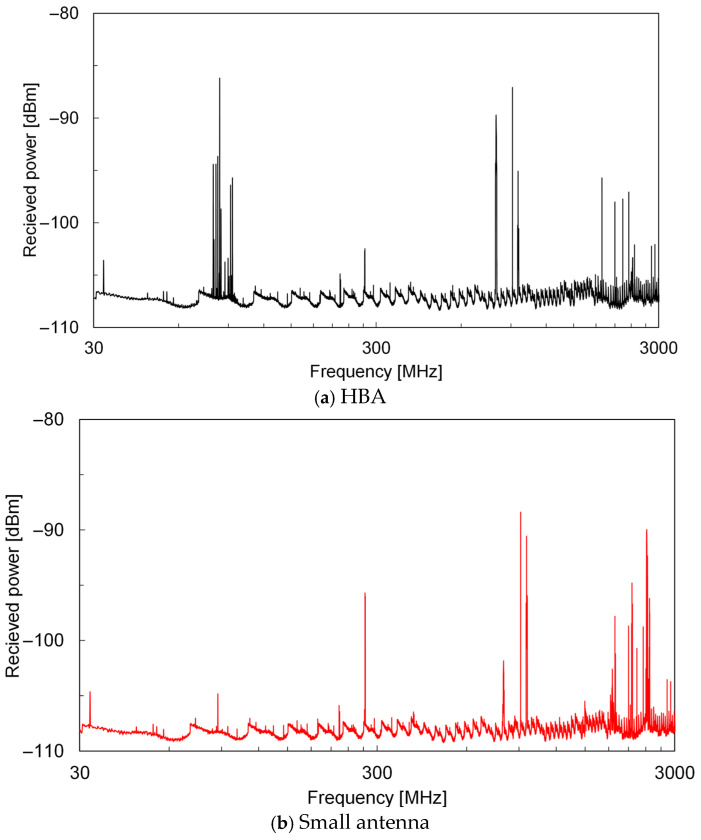
Received power spectrum of the antennas in an outdoor environment.

Figure 11a shows the frequency spectrum of the power received by the HBA. For comparison, the spectrum received by the small 2.4 GHz whip antenna (W1095, Pulse Electronics, Inc., San Diego, CA, USA) is shown in Figure 11b. The HBA demonstrated excellent reception of low-frequency AEMWs, such as FM radio broadcast waves near 80 MHz. At higher frequencies, such as near 2.4 GHz, the HBA exhibited reception power characteristics comparable to those of the small antenna specifically designed for that frequency band. This is because, as shown in Section 3.3, the input impedance of the HBA as viewed from the interface electrode attached to the wrist was well matched over a wide frequency range, ensuring favorable reflection characteristics. Additionally, as discussed in Section 3.1, while the radiation resistance and gain of the electrically small antenna decreased, the electrically large HBA, even with attenuation and shielding by the body’s water content, maintained sufficient gain. These results suggest that the HBA exhibits a superior reception power at both low- and high-frequency bands.

The total harvested power *P*_sum_ at each location, as determined from the results shown in Figure 11 and using Equation (6), was −31.9 dBm (0.64 μW) for the HBA and −33.5 dBm (0.45 μW) for the small antenna. The fact that the *P*_sum_ of the HBA exceeded that of the small antenna by 1.6 dB was due to the HBA’s excellent reception of AEMWs with a high power density near 80 MHz. Although −32 dBm is not a large amount of harvested power, it is sufficient for the continuous or intermittent operation of devices such as temperature sensors, accelerometers, Hall sensors, and low-power microcontrollers. In addition to driving the sensors themselves, a power supply for wireless communication circuits is required for coordination among sensors and data transmission to hub devices. For example, the power consumption for each Bluetooth Low Energy advertising event is typically about 50–200 μJ, depending on the number of channels and the payload length [45]. Therefore, if a harvested power of approximately −30 dBm can be secured, intermittent communication at intervals of several seconds to several minutes is possible. Furthermore, combining the system with power-management ICs that can operate at an input power below −30 dBm enables efficient energy buffering and advanced power management such as maximum power point tracking [46]. These results show that, compared to small antennas that can be implemented in wearable devices, an HBA can increase the amount of power harvested from AEMWs and can sufficiently drive various sensors and wireless modules for wearable devices.

Moreover, while the volume of the small antenna (excluding the connector) is 2086 mm^3^, the interface electrode for the HBA (excluding the connector) is only approximately 41 mm^3^, resulting in a size reduction of more than 1/50 in terms of the antenna volume required for energy harvesting implementation. Although additional factors beyond simple volume comparison should be taken into account when considering integration into actual wearable devices, the ability of the HBA to achieve substantial miniaturization while improving the harvested power remains a significant advantage.

### 4.3. Human Safety Evaluation

**Figure 12:**
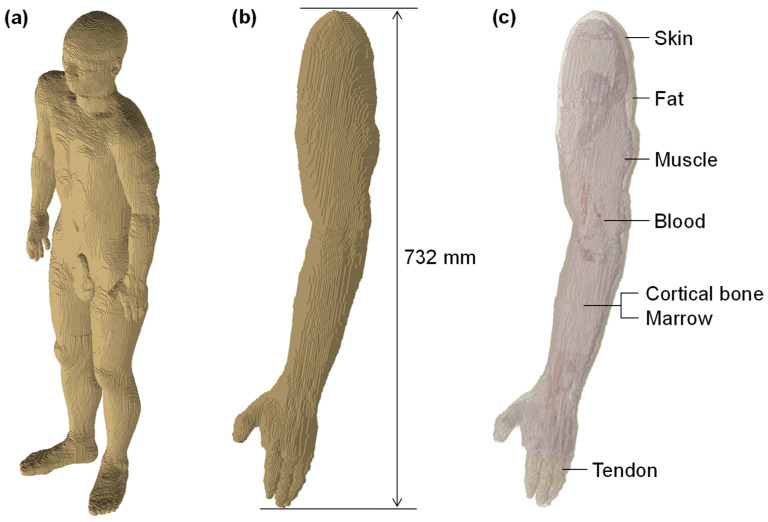
Overview of the human model. (**a**) Whole-body, (**b**) arm portion, and (**c**) tissue composition.

In AEMW energy harvesting, no intentionally high-power electromagnetic waves are irradiated to the human body; the HBA simply receives AEMWs emitted from systems that comply with legal regulations regarding their frequency and transmitted power. Thus, the exposure experienced by the human body is no different from that of the general public in everyday situations, and the risk of electromagnetic exposure is extremely limited. However, when harvesting power at the interface electrode of an HBA, it is possible that the current could be concentrated near the electrode, potentially increasing the local exposure level. Therefore, we evaluated the risk of electromagnetic exposure with an electromagnetic field analysis using the arm portion of the anatomically detailed numerical human model TARO [47], which consists of multiple biological tissues. Figure 12a shows the overview of the whole-body human model, (b) shows the extracted arm portion, and (c) depicts the tissue composition of the arm. The arm model includes seven types of biological tissues: skin, fat, muscle, blood, cortical bone, cancellous bone, and tendon.

In this study, the exposure risk was evaluated in terms of the specific absorption rate (SAR) around the interface electrode under actual power-harvesting conditions. SAR is an index representing the rate at which biological tissues absorb high-frequency energy. SAR is defined as follows:

(7) $$SAR=\frac{\sigma E^{2}}{\rho }$$

where *σ* is the electrical conductivity of the tissue, *E* is the root-mean-square value of the electric field induced in the tissue, and ρ is the mass density of the tissue. For example, when 0.64 μW of power is harvested by an HBA in an outdoor environment, and considering a load impedance of 50 Ω, the voltage generated between the electrodes is estimated to be approximately 5.7 mV. In this case, the maximum 10 g average SAR in the skin tissue directly beneath the electrode was calculated to be 1.4 × 10^−3^ W/kg. According to the guidelines established by the International Commission on Non-Ionizing Radiation Protection, the 10 g average SAR limit is 2.0 W/kg [48]; thus, the SAR under the assumed conditions was less than 1/1000 of the limit, demonstrating sufficient safety from the viewpoint of electromagnetic exposure. Therefore, AEMW energy harvesting using an HBA is considered to be a sufficiently safe technology in terms of the exposure risk to the human body.

## 5. Conclusions

In this study, we worked on improving the performance of ambient electromagnetic wave (AEMW) energy harvesting using a human body antenna (HBA), which utilizes the human body as a large receiving element for electromagnetic waves, for powering sensors in wearable healthcare systems. First, the power density of the AEMWs was measured using a standard antenna over a frequency range of 30 MHz–3 GHz in indoor, outdoor, and basement environments on a university campus. The results confirmed that, except for special propagation environments such as basements with significant electromagnetic shielding, AEMWs derived from various wireless systems exist over a wide frequency range, both indoors and outdoors. The fact that a certain level of AEMWs exists regardless of the environment of the sensor or user is an extremely advantageous feature for energy harvesting in healthcare systems that continuously monitor the user’s health status over long periods. Next, we prototyped a flexible HBA–circuit interface electrode for collecting power received by the HBA and evaluated its reflection characteristics when attached to the body. The prototype electrode achieved a reflection coefficient below −10 dB at 30–350 MHz and below −6 dB for most of the 350 MHz–3 GHz range, confirming excellent reflection characteristics over a wide bandwidth. Subsequent experiments using a simulated AEMW source revealed that the HBA exhibited much better reception characteristics than the small whip antenna, particularly in frequency bands below 300 MHz. On the other hand, at frequencies above 300 MHz, the reception characteristics of the HBA tended to deteriorate due to the absorption of the received electromagnetic waves by the water content in the body. Finally, the amount of power harvested by the HBA was measured under actual environmental conditions and estimated to be −31.9 dBm. This value exceeded the power of the small antenna under the same conditions (−33.5 dBm) by 1.6 dB, making it sufficient for the intermittent operation of low-power wearable sensors and Bluetooth Low Energy modules. In addition, human safety was evaluated through electromagnetic field analysis using a numerical human model, and the results confirmed that the specific absorption rate under actual power harvesting conditions is well below international safety limits. Therefore, the proposed AEMW energy harvesting using an HBA is considered to be sufficiently safe in terms of electromagnetic exposure to the human body. These results demonstrate that AEMW energy harvesting using an HBA enables a constant supply of harvested power, regardless of location, and is a promising approach for building next-generation wearable healthcare systems. This study is a pilot study based on experiments with a limited number of subjects. In the future, it will be necessary to optimize the system based on reproducibility and a statistical analysis involving more diverse subjects and additional environments. It will also be essential to evaluate the effects of factors such as the mounting position, posture, clothing, and environmental humidity, as well as to assess the safety of the long-term use of the interface electrode. In addition, we plan to further develop the theoretical framework of the HBA using an electromagnetic field analysis with a whole-body numerical human model and optimize the electrode interface, considering the target frequency range and wearable sensor mounting position, with the goal of further increasing the amount of harvested power.

## Data Availability

The original contributions presented in this study are included in the article. Further inquiries can be directed to the corresponding author.
